# Anti-Aging Effect of Traditional Plant-Based Food: An Overview

**DOI:** 10.3390/foods13233785

**Published:** 2024-11-25

**Authors:** Gitishree Das, Srinivasan Kameswaran, Bellamkonda Ramesh, Manjunatha Bangeppagari, Rajat Nath, Anupam Das Talukdar, Han-Seung Shin, Jayanta Kumar Patra

**Affiliations:** 1 Research Institute of Integrative Life Sciences, Dongguk University-Seoul, Goyang-si 10326, Republic of Korea; 2 Department of Botany, Vikrama Simhapuri University College, Kavali 524201, Andhra Pradesh, India; 3 Department of Biochemistry and Molecular Biology, University of Nebraska Medical Center, Omaha, NE 68198, USA; 4 Department of Cell Biology and Molecular Genetics, Sri DevarajUrs Academy of Higher Education and Research (A Deemed to Be University), Tamaka, Kolar 563103, Karnataka, India; 5 Department of Life Science and Bioinformatics, Assam University, Silchar 788011, Assam, India; 6 Department of Biotechnology and Microbiology, School of Natural Sciences, Techno India University, Agartala 799004, Tripura, India; 7 Department of Food Science and Biotechnology, Dongguk University-Seoul, Goyang-si 10326, Republic of Korea

**Keywords:** aging, nutraceuticals, oxidative stress, antioxidants, plant-based supplements

## Abstract

Aging is a complex process that involves many physiological mechanisms that gradually impair normal cellular and tissue function and make us more susceptible to diseases and death. It is influenced by intrinsic factors like cellular function and extrinsic factors like pollution and UV radiation. Recent scientific studies show that traditional plant-based foods and supplements can help mitigate the effects of aging. Nutraceuticals, which are dietary supplements with medicinal properties, have gained attention for their ability to prevent chronic and age-related diseases. Antioxidants like flavonoids, carotenoids, ascorbic acid, terpenes, tannins, saponins, alkaloids, minerals, etc. found in plants are key to managing oxidative stress, which is a major cause of aging. Well-known plant-based supplements from *Bacopa monnieri*, *Curcuma longa*, *Emblica officinalis*, *Ginkgo biloba*, *Glycyrrhiza glabra*, and *Panax ginseng* have been found to possess medicinal properties. These supplements have been shown to improve cognitive function, reduce oxidative stress, improve overall health, and potentially extend life and enhance the excellence of life. The obtained benefits from these plant species are due to the presence of their bioactive secondary metabolites, such as bacosides in *Bacopa monnieri*, curcumin in *Curcuma longa*, ginsenosides in *Panax ginseng*, and many more. These compounds not only protect against free radical damage but also modulate key biological pathways of aging. Also, traditional fermented foods (tempeh and kimchi), which are rich in probiotics and bioactive compounds, support gut health, boost immune function, and have anti-aging properties. The molecular mechanisms behind these benefits are the activation of nutrient-sensing pathways like AMPK, SIRT/NAD+, and mTOR, which are important for cellular homeostasis and longevity. This review shows the potential of traditional plant-based foods and dietary supplements for healthy aging, and more studies are needed to prove their efficacy and safety in humans. Incorporating these natural products into our diet may be a practical and effective way to counteract the effects of aging and overall well-being. The foremost goal of this review is to emphasize the importance of supporting the body’s antioxidant system by consuming the right balance of natural ingredients in the diet.

## 1. Introduction

**Figure 1:**
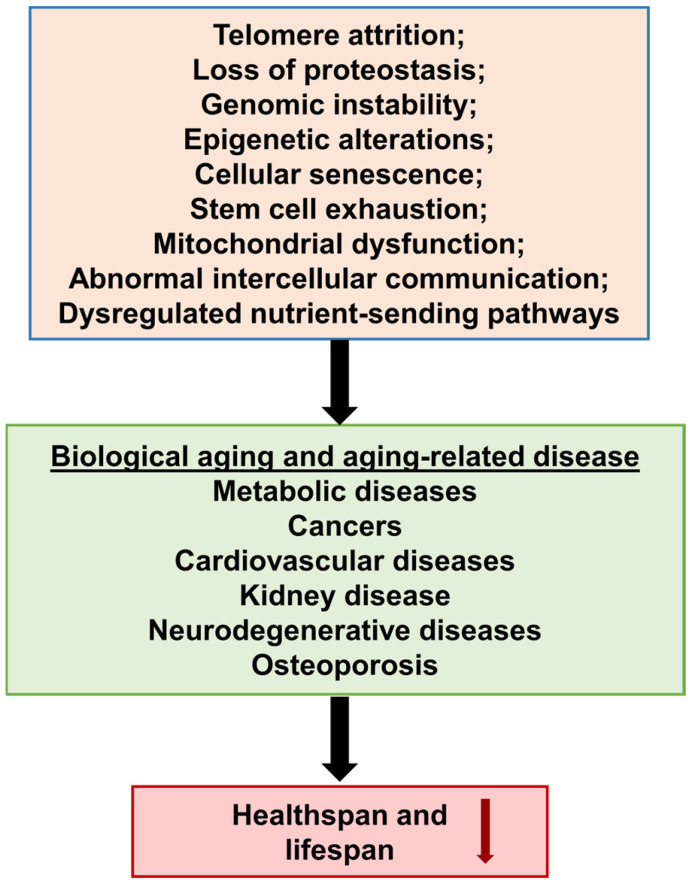
Signs and diseases associated with aging.

The ongoing physiological mechanism of aging interrupts the normal roles of “tissues and cells”, increasing the risk of illness and demise (Figure 1). This appears to be a normal course of growth and aging. Aging is intricate and is impacted by both external and internal factors [1]. External variables such as air pollution (smoking and dust), prolonged sunlight exposure, hormone imbalances, malnutrition, and harmful ultraviolet (UV) rays affect cell function. Internal variables include normal biological functions that occur in a cell [2]. With aging, the cardiac, lung, and renal systems deteriorate [3]. In addition, a sedentary lifestyle and malnutrition increase the risk of fractures, overweight, heart problems, arthritis, diabetes, type 2 diabetes, cancer, and high blood pressure in elderly people [4]. Wrinkles indicate aging of the skin, which can be prevented by taking supplements with significant antioxidants, eating healthy foods, and applying skincare products [5]. The adverse consequences of free radicals may be mitigated by taking these actions [6].

Many studies have recently been conducted on the impact of nutrition on animal and human aging [7]. Nutraceutical products, including nutritional foods, dietary additives, and botanical extracts, can be used as nutritional supplements to improve health over time [8]. According to experts, antioxidants may benefit either chronic or age-associated illnesses, particularly carcinomas, as well as neurological disorders [9]. Flavonoids, carotenoids, ascorbic acid, terpenes, tannins, saponins, alkaloids, minerals, etc. are nutritional supplements that have the potential to act as antioxidants [10,11,12]. They help avoid and manage chronic illnesses that are linked to reactive oxygen species (ROSs), which leads to healthy people living longer [9]. Supplemental nutrients have positive effects on the gastrointestinal and immunological systems but can have negative effects on inflammation and degenerative alterations in the human body [13]. Therefore, they improved their standard of living. Considering this, in the current review, the potential of traditional plant-based foods and dietary supplements is discussed concerning their beneficial effect on aging and related ailments. The foremost goal of this review is to emphasize the importance of supporting the body’s antioxidant system by consuming the right balance of natural ingredients in the diet.

## 2. Plant-Based Supplements with Potential to Prevent Aging

It is well known that plants and their component elements, like vitamins, flavonoids, and carotenoids, have “antioxidant potentials” that might help “prevent and treat chronic” diseases caused by reactive oxygen species (ROSs) [14]. These nutrients have antagonistic reactions towards human inflammation and degeneration systems, strengthening the immune system and improving digestion in the process [15]. Adaptogens are extracted from medicinal plants and used to stabilize human biological processes and preserve homeostasis [16]. These are synthetic compounds like aphobazole, bemethyl, levamisole, bromantane, etc. Naturally occurring adaptogens or plant adaptogens are extracts from *Rhaponticum carthamoides*, *P. ginseng*, *Eleutherococcus senticosus*, *Schisandra chinensis*, *Rhodiola rosea*, etc. [17,18,19,20]. Adaptogen normally acts on the adrenal, amygdala, hypothalamus, pituitary, and thyroid glands. In the case of stress and fatigue, the nor-adrenaline becomes imbalanced; hence, the adaptogens trigger the hormonal response to manufacture and discharge more adrenaline and nor-adrenaline hormones, thereby helping the body to respond more efficiently and proficiently to the additional hormones and shut down more rapidly [21]. The body is better able to withstand damage from other risk factors because these substances reduce the sensitivity of cells to stress [22]. Additionally, they support the restoration and enhancement of regular physiological function [23]. The following section discusses several well-known adaptogens and the most popular plant-based supplements. In an article, Singh et al. [23] have listed a number of plant biomolecules that are used as adaptogens (Table 1).

Reproduced from Singh et al. [23], distributed under the terms of the Creative Commons Attribution License, which permits unrestricted use, distribution, and reproduction in any medium (Original source Table 1).

There are reports that, due to their chemical nature, the antioxidants are able to neutralize the ROS and also reduce the pro-inflammatory Th1-type cytokine cascades and associated symptoms [72,73]. But with the passage of time, the above conclusion has become vague when the food sector industries started adding more vitamins to the food products in the name of preservatives and colorants without considering their side effects. Usually, most of the added compounds include vitamin C and sodium sulfite, which are strong antioxidants. Reports say that excessive use of these additives in the foods gave rise to increased allergy and asthma [74]. Hence, it is concluded that excessive use of antioxidants resulted in the antioxidative stress that plays a major role in the development of allergies [75].

### 2.1. Plants

#### 2.1.1. *Bacopa monnieri* (L.) Wettst

*B. monnieri* is an annual plant that occasionally goes by the name “brahmi” [76]. It had tiny rectangular leaves and purple blossoms. This therapeutic herb encompasses extremely valued nootropic composites, namely, bacosides [77]. “Brahmine and herpestine” have been both main metabolites found predominantly obtained from this plant [78]. Phytochemicals found in brahmi help learning and mental health by protecting brain cells from the detrimental impacts of “free radicals” [79]. In their “in vitro” and “in vivo” studies, Shinomol et al. used extracts from *B. monnieri* plant and “3-nitro propionic acid” (“NPA”), a mycotoxin that is toxic to both animals and humans. A substance derived from *Bacopa monnieri* was found to have been very helpful in both preserving the oxidative processes generated by “NPA” and reducing the levels of “thiol and glutathione. According to studies, “NPA” was found to be efficient in reducing “oxidative stress” in the mitochondria and dopaminergic (N27) cells in the striatum of rats [80]. A week-long “placebo-controlled” Kumar and associates carried out research to find out how “*Bacopa monnieri* extract” affected the mental faculties of medical students. The research outcomes indicated that students’ cognitive performance improved significantly [81].

#### 2.1.2. *Curcuma longa* L.

A chemical known as “curcumin” is produced by the species “*Curcuma longa* L.” [82]. Numerous “biological effects”, like anti-inflammatory, “anti-cancer”, and antioxidant properties, are well known [83]. Curcumin has the potential to be used as a medicinal agent to treat various cancer types owing to its inherent qualities [84]. Copious studies have established that curcumin destroys the action or function of “pro-inflammatory cytokines”, such as “tumor necrosis factor-α” (TNF-α), “prostaglandin E2” (PGE2), and “cyclooxygenase-2” (COX-2) [85]. The antioxidant properties of curcumin allow it to scavenge oxidants and prevent lipid peroxidation [86]. Oral curcumin therapy is being demonstrated to “halt tumor growth” and minimize “cystic fibrosis” in animals, while human research is still ongoing [84]. A study found that curcumin triggers redox communication along with the “phosphatidylinositol 3-kinase”/“Akt” (“protein kinase B; PKB”) mechanisms that lead to a “cell stress response” in “human fibroblasts”. This shows that cellular antioxidant defenses activated by curcumin can be a useful strategy for anti-aging interventions [87].

Furthermore, research has demonstrated that it lengthens the lifetime of “fruit flies”, “nematodes”, and “mice” [88,89,90]. Research indicates that curcumin enhances age-related disease indicators, including cancer, diabetes, and atherosclerosis [91]. Furthermore, it is demonstrated that “curcumin” shields women with cancer against “radiation and chemotherapy-induced” dermatitis [92,93]. Because “curcumin” can delay “cellular senescence”, it can be demonstrated through various investigations to possess “anti-aging” characteristics [94]. “Cox et al.” assessed the impacts “of solid lipids”, such as “curcumin”, in both physical and mental health in healthy persons aged “60–85”. “Acute” (“1 and 3 h after a single dose”), “chronic” (“4 weeks”), and “acute-on-chronic” (after starting regular therapy, take a single dose every 1 to 3 h) responses to 400 mg of Longvida^®^, a solid–lipid curcumin formulation, were evaluated in this study. Working memory significantly improved with both chronic and acute dosages, according to the data. It lowered total and LDL cholesterol as well as physical weariness, as assessed by the Chalder Weariness Scale [95].

#### 2.1.3. *Emblica officinalis* Gaertn. (Synonym of *Phyllanthus emblica* L.)

The plant known as amla or *Emblica officinalis* represents the Phyllanthaceae family [96]. These plants have been reported to be a rich source of vitamin C, minerals, and other types of bioactive compounds such as flavonoids, tannins, terpenoids, alkaloids, etc. [97]. Flavonoids (quercetin) and alkaloids (phyllantine and phyllantidine) are reported in this plant [97]. These are widely recognized as “Amla churn” and are reported to lower the cholesterol and enhance memory [98]. Both the body and brain cholesterol levels can be effectively lowered by including amla in the diet [99]. Moreover, it has been mentioned that it is a helpful functional diet for Alzheimers disease treatment [100]. To evaluate the “skin-lightening” effectiveness of an oral cosmetic mixture containing “glycolic acid”, kojic acid, and an extract from *E. officinalis*, Draelos et al. conducted a double-blind trial. Researchers discovered that their “topical” preparation performed 4% better than hydroquinone, leading them to propose it as a potential natural replacement for light to mild “facial dyschromia” [101]. Additionally, in addition to its “antioxidant” qualities, isolation of “*E. officinalis*” inhibits “GABA” and “MAO-A” in mouse tests, exhibiting “antidepressant” capabilities [102].

#### 2.1.4. *Ginkgo biloba* L.

*G. biloba*, also referred to as gingko, increases the amount of oxygen available in the tissues [103]. “*Ginkgo* leaves” have been reported to considerably sustain blood flow and blood sugar levels inside brain tissue [104]. In addition, it enhances the brain activity related to mental processes [105]. When obtained from “ginkgo leaves”, “flavone glycosides” such as “ascorbate”, “shikimate”, isorhamnetin, and “lactone derivatives” (“ginkgolides”) are produced. These compounds are potent antioxidants that combat “free radicals” [106]. Huang has carried out research to evaluate the “*Gingko biloba*” component’s effect “on the liver” functions of aged rats. Therefore, it is confirmed that “*G. biloba*” extract is used to treat oxidative stress; the stress is reduced by raising SOD activity and decreasing “liver metalloproteinase” and “malondialdehyde” amounts [107]. *G. biloba* extract administration has been shown in another investigation to enhance cognitive performance in old female rats [108]. To determine how well “*G. biloba*” extract works for managing “Alzheimers disease” as well as mental wellness, a clinical investigation is being conducted. The consumption of *G. biloba* extract has been shown through extensive investigation to enhance cognitive functioning in patients with moderate dementia [109].

#### 2.1.5. *Glycyrrhiza glabra* L.

Member of the Fabaceae family, *G. glabra* is a plant often known as licorice [110]. The roots and rhizomes of this plant have been shown to have calming effects on the brain and help control glucose levels in the body [111]. The primary physiologically active ingredient in this “antioxidant-rich” plant, glycyrrhizin, preserves healthy “nervous system” function, enhances memories, and shields brain cells from “oxidative damage” [112]. Because of its potential antioxidant qualities, “*G. glabra*” includes a “phenolic” molecule called licorice that may be useful in “chelating metal ions” as well as eliminating “free radicals” [113]. Scopolamine-induced Alzheimer in mice is said to be ameliorated in the brain by “*G. glabra*” [114]. Moreover, “Dhingra and associates” documented enhancements in the cognitive abilities of rats administered “*G. glabra*”. Various dosages of “*G. glabra*” extract were used over a week in a row. Studies have shown that memory enhancement in a mouse model was achievable “at 150 mg/kg” [115].

#### 2.1.6. *Panax ginseng* C.A.Mey

The medicinal properties of “*P. ginseng*” and ginseng are well known [104,116]. Ginsenoside is an active ingredient produced by plant roots [117]. In addition to impacting the immune system, this bioactive substance also affects the body’s response to “anxiety”, “weakness”, “nervousness”, and “trauma” [118]. It also improves “memory” and “learning” and has anti-stress properties [116]. According to research, administering ginseng to young mice with leukemia extended their life expectancy [119]. By lessening oxidative stress, *Panax ginseng* has been shown in another investigation to enhance antioxidant activity and reduce lipid peroxidation [120].

Additionally, double-blind clinical research has verified that ginseng consumption enhances people’s psychomotor performance [121]. Further research indicates that “*P. ginseng*” may possess “anti-melanogenic” properties and is connected with the stimulation of the foxo3a gene, which is recognized as a factor in life expectancy [122]. Specific investigations have claimed that *P. ginseng* can prevent skin aging. Furthermore, “double-blind”, “randomized”, and “placebo-controlled” investigations were carried out to assess “ginsenosides” and “*Panax ginseng*’s” potential to postpone the cutaneous aging process. The results of the trial showed a notable decrease in the appearance of wrinkles, and the medication had no adverse effects on any of the subjects [123].

### 2.2. Prepared Foods

#### 2.2.1. Tempeh

Many Indonesians appreciate tempeh, an ancient fermenting soybean food, as an affordable and wholesome source of protein. It is prepared by inoculating cooked, soaked, and dehulled soybeans with Rhizopus species [124]. The health benefits and anti-aging qualities of tempeh have just caught the attention of many researchers. In female rats before or after menopause, tempeh extract has been shown to have anti-aging effects by preserving uterine quality, improving skin, and strengthening bone structure simultaneously [125]. Tempeh extract supplementation exhibited comparable anti-aging effects to commercial hormone therapy (including genistein, ethinylestradiol, and somatotropin). This enhancement can be attributed to the presence of soy isoflavones, which are enriched through fermentation. In rats after menopause, these isoflavones have been shown to restore estrogen levels, which may improve the quality of life for postmenopausal women [125].

#### 2.2.2. Kimchi

Kimchi, a traditional Korean fermented dish primarily made from cabbage, is renowned not only for its tangy taste but also for its potential anti-aging properties [126]. Packed with probiotics, vitamins, and antioxidants, such as vitamins A and C, kimchi helps promote gut health and bolster the immune system [127]. Furthermore, the fermentation process produces beneficial bacteria like *Lactobacillus*, which aids in digestion and may contribute to overall longevity [126]. Furthermore, ingredients in kimchi, such as isothiocyanates, have demonstrated anti-inflammatory and anti-cancer properties, potentially slowing down the aging process [128,129]. Regular consumption of kimchi as part of a balanced diet may thus offer a flavorful and natural means of promoting vitality and wellness.

#### 2.2.3. Chungkookjang

Chungkookjang, a traditional Korean fermented soybean paste, has garnered attention for its potential anti-aging properties. Rich in antioxidants, such as polyphenols and isoflavones, Chungkookjang is believed to combat oxidative stress, a key contributor to aging [130,131]. Moreover, its fermentation process enhances the bioavailability of nutrients, including vitamins and minerals, which are vital for maintaining skin health and vitality [132]. Additionally, Chungkookjang contains probiotics, beneficial bacteria that promote gut health and may indirectly influence skin aging through their impact on systemic inflammation and immune function [133]. With its multifaceted approach to combating aging, Chungkookjang stands as a promising candidate in the pursuit of youthful longevity.

## 3. Plant-Based Metabolites with Healthy and Restorative Effects

A number of bioactive compounds with numerous healthy and restorative properties are extracted from several plants, and a group of the most potent plant-based metabolites with anti-aging effects, which are consumed as food, is discussed below.

### 3.1. Polyphenols

Most phytochemicals, especially “polyphenolic” substances, have been synthesized by “plants” and can be found in different diets and beverages, among other “fruits”, “vegetables”, cereals, and drinks [134]. Researchers from all over the world have been interested in polyphenols because of their inherent qualities, including their ability to act as antioxidants along with their “anti-inflammatory” and anticarcinogenic activities [135]. Many “polyphenolic” compounds are being studied, such as “resveratrol”, “proanthocyanins”, and “silymarin”. The effectiveness of these treatments in experimental animals subjected to “UV-induced oxidative stress”, “skin irritation”, and “DNA damage” [136]. When used with sun protection products, these polyphenols also protect “the skin” against “UV radiation”-mediated dermatological problems, which helps to lower the possibility of “skin tumors” [137]. Certain “polyphenols” with possible health benefits are listed below.

The natural “polyphenolic” compound known as “stilbenes”, or “resveratrol”, is found in “the outer layers” of grapes and groundnuts and may have antioxidant properties [138]. It has become the focus of many investigations in recent decades because it utilizes an “anti-aging” substance [139]. It can also function as an antioxidant and has anti-inflammatory and radical-scavenging qualities [140]. Research has shown that it works well for managing a variety of ailments, including heart disease and Alzheimers disease [141]. In addition, it has protective properties on the skin of humans, as demonstrated by a study performed on HaCat cells that were treated with sodium nitroprusside, a source of nitric oxide free radical [142]. A study evaluating the effects of “resveratrol” using proliferation and repression of elastin activities was carried out “in vitro on skin fibroblasts”. Findings showed an increase in dose in the amount of proliferating cells and a notable suppression of collagenase activity [143]. There is a dearth of evidence in human beings supporting the idea that resveratrol can prevent aging at the cell level and could be an advancement in geriatric and anti-aging treatment [144]. Long recognized for its ability to affect mitochondrial metabolism, “resveratrol” further delays aging and prevents chronic illnesses by upregulating “PGC-1α”, a receptor that activates peroxisome proliferator-activated receptors [145,146].

Apples are a rich source of polyphenolic chemicals, including epicatechin, rutin, phloretin, chlorogenic acid, and proanthocyanidin B2 [147]. Apples can help stop “low-density lipoprotein (LDL)” from oxidizing, according to various research [148]. The effect of apple polyphenols on the activity in the genes encoding catalase, CAT (methuselah), “cytochrome c oxidase subunits III”, Rpn11, SOD, “VIb”, and CAT (catalase) was investigated. The results of the study demonstrated that drosophila lived ten percent more time when exposed to “apple polyphenols”. It was also shown that the fruit flies had downregulated Mth, elevated “CAT”, “SOD1”, and “SOD2”, and that there had been little alteration within the functioning of the “CcO subunits”, “Rpn11”, or “VIb genes” [149]. Furthermore, studies on mice with genetic impairments and those with normal aging have shown that condensed juice from apples has “neuroprotective” qualities. But the precise makeup of Apple’s anti-aging properties and underlying processes remains a mystery [150].

Berries contain larger amounts of “polyphenols” compared with other vegetables and fruit [151]. Reduced signs of aging are linked to “blueberry extract’s” strong “antioxidant” activity [152]. It has been reported that consuming blueberry extract reduces functional and physiologic deficits associated with aging [153]. Researchers who studied how the short-term supplementation with blueberries could affect the brain’s ability discovered that giving rats blueberry extract supplements might result in improved heat shock protein 70-mediated protection against a number of neurodegenerative processes in the brain of rats [154]. To comprehend the fundamental process, blueberry extracts’ capacity to extend life has additionally been looked into in fruit flies. The investigation’s findings indicated that drosophila’s life expectancy was significantly increased by about 10 percent when 5 mg/mL blueberry extract was incorporated into their diet [155].

### 3.2. Flavonoids (Phlorizin)

It was recently discovered that just a handful of plants can synthesize phlorizin [156]. It has been used as an opportunity to assess physiological functioning and has been heavily utilized by the pharmaceutical industry for over a century [136]. Phlorizin’s nutritional advantages have been the subject of numerous investigations. Using murine senile osteoporosis models, phlorizin and phloretin’s anti-aging properties were examined in the latest study. The investigation revealed that phlorizin regulates the activation of nuclear factor “kappa-B ligand” to “osteoprotegerin”, a biological indicator of “osteoporosis”. Furthermore, phenolphthalein reduced the number of osteoclast cells expressing “tartrate-resistant acid phosphatase” [157]. There is an enormous amount of “phenolizin” in immature apples. Pilot studies on humans have demonstrated the potential benefit of “phenolizin-containing” immature apples in lowering hyperglycemia after eating. Six participants in good health participated in the research and discovered that consuming immature apples significantly reduced the postprandial blood sugar response and increased urine sugar [158]. The results of the mixtures involving the eight different herb extracts—turmeric, “mulberry leaf”, apple, “elderberry”, white bean, and mulberry fruit—on “postprandial insulin” and blood sugar response were examined. Mela and colleagues carried out the study. The study’s conclusions, which showed that hyperglycemia accelerates aging, emphasize phlorizin’s capacity to reduce aging and improve “quality of life” [159]. Reliability as an abundant supplier of compounds with “antioxidant” activity has been demonstrated by several “plant extracts” [160]. Skin aging symptoms have been demonstrated to be positively impacted by metabolites such as apigenin, genistein, and silymarin [103]. Clinical or human research has not yet revealed phenorizin’s full “anti-aging potential”.

### 3.3. Tea Catechins and Theaflavins

The health benefits linked to tea drinking can be ascribed to two of its natural constituents, namely, flavonoids and catechins [161]. Research has shown that consuming “green or black tea” daily can stop “DNA” molecules from oxidizing [162]. Numerous studies have demonstrated that consuming “tea polyphenols” orally and applying “topical green tea” therapy can protect animals from developing skin cancer caused by chemicals or ultraviolet (UV) rays [163]. Tea’s “flavonoids” and “catechins” are “anti-inflammatory” and “anticarcinogenic” substances [161]. Elmets, with his severe sunburn, says “green tea extract or one of its” constituents was originally put to willing participants’ skin. The specified areas were then subjected to two low-erythema treatments using solar-simulated radiation. Subsequently, the “skin” was inspected to determine whether UV-induced DNA damage had any biochemical, clinical, or histologic features. The findings showed that the erythema response brought on by UV radiation is inhibited by tea extract in a dose-dependent manner. Additionally, the histologic investigation revealed fewer Langerhans and sunburn cells [164].

Furthermore, epidermal DNA damage was decreased by tea polyphenol extracts. The findings suggest that “tea polyphenol may therefore act to be a natural remedy to photoprotection [164]. Chiu and colleagues [165] examined the impact of orally and topically administered green tea pills on the histological and medical characteristics of photoaging. Forty women by acceptable photoaging took part in the current trial, as well as selecting a treatment or a placebo or “300 mg of oral tea” supplements two times daily “for eight weeks”, along with 10 percent “green tea cream”. The results of the investigation showed no discernible differences in the clinical photoaging traits in both green tea-treated and placebo groups. However, the number of “elastic tissues” in those treated was increased histologically [165].

### 3.4. Black Rice Anthocyanins

Antioxidants, which are plentiful in black rice, are being shown to alleviate Alzheimers disease symptoms in patients [9]. It also possesses anti-inflammatory and anticarcinogenic properties [166]. It also contains two pigments in plenty, *Peonidin 3*-O-*glucoside* chloride and “cyanidin-3-o glucoside (chloride)” [167]. Based on studies by Zuo et al. [168], “black rice” may extend the life of drosophila. To make a decision, the effects upon gene activities of catalase, methuselah, “Rpn11”, superoxide dismutase 1, and superoxide dismutase 2. According to the experiment’s findings, giving Drosophila “30 mg/dL” of “black rice anthocyanins” extended their longevity by 14 percent. Furthermore, observed were the elevated gene activities of superoxide dismutase 2, superoxide dismutase 1, “Rpn11”, and catalase and the decreased gene activity of methuselah [168]. In research employing an account of chronic aging in rats, Huang et al. demonstrated the “anti-aging”, “anti-fatigue”, and “anti-hypoxic” properties of black rice anthocyanins [169].

### 3.5. Carotene

It has been established that two carotenes that are derived from “vitamin A”, namely, “lycopene” and “β-carotene”, have potent antioxidant and photoprotective qualities [170]. Besides, astaxanthin has also been found to be effective in reducing the aging in the skin, followed by increasing the moisture content and reducing the wrinkles [171]. Carotenoids exercise their properties by a number of mechanisms of action [172]. Usually, these compounds are obtained from foods and are absorbed into the body and are accumulated in the skin and act as a protectant against sunburn, UV radiation, and damages to the skin from external environmental factors [172,173,174]. The anti-aging property of carotenoids is primarily established on their promotion of nuclear factor erythroid 2-related factor 2 migration into the nucleus followed by the transcription of antioxidants and detoxification of enzymes [175]. In addition, Bakac et al. has also studied the carotenoid types such as astaxanthin, β-carotene, lycopene, and zeaxanthin on some aging-related diseases [176]. The author concluded that carotenoid supplementation is beneficial in checking and delaying the aging and aging-related diseases, averting and treating eye fatigue and dry eye disease, and enhancing macular function [176]. “Lycopene” plus “β-carotene” can result in a little improvement in the appearance of skin [177]. Plant-based foods such as papaya, “carrots”, “mangoes”, and “pumpkins” are rich sources of β-carotene [178]. It gained importance as a carotenoid due to its attributes, which include “pro-vitamin A activity”, the ability to fight “lipid radicals”, and the ability to quench “single oxygen” [179]. It has been observed that “β-carotene” has good light-protective qualities and can avoid ultraviolet-induced “erythema” [180]. Research has shown a link between biological aging and reduced levels of beta-carotene in the blood plasma. β-carotene may influence telomerase activity in older persons, according to a study that involved 68 elderly participants [181].

“*Lycopene*”, “red carotene”, “carotenoid”, as well as phytochemicals, are found in papayas, tomatoes, watermelons, carrots, and a variety of other colored fruits and vegetables [5]. Its potent ability to quench singlet oxygen is despite an absence of “vitamin A” activity [182]. Furthermore, lycopene’s function in reducing oxidative damage to tissues was validated by research. Research revealed that “skin lycopene” is significantly more susceptible to UV radiation degradation than “β-carotene” [183]. Moreover, lycopene-containing compounds were additionally demonstrated to be incredibly efficient over malignant cells and to dramatically lower “MMP-1” action, and this is linked to the degradation of “collagen” [184]. The two main “carotenoids” present in human muscles along with blood, “lycopene” as well as “β-carotene”, are said to control the characteristics of the skin [185]. Cheng et al. [186] discovered in recent research that “lycopene” stimulates the A549 cells’ early incision-repairing mechanism in vitro. The current study has demonstrated a “molecular mechanism” that demands both animal and in vivo research [186].

### 3.6. Vitamins

“Ascorbic acid”, alternative term for “vitamin C”, is a vitamin soluble in water [187]. Because of its powerful reducing capabilities, this colorless molecule has a great deal of potential as an antioxidant [188]. Water-loving plants are the ideal habitat to grow light-sensitive “ascorbic acid” [189]. These crystal compounds need to be ingested through everyday food because mammals are unable to synthesize them [190]. To prevent illnesses associated with low vitamin C, such as scurvy and heart problems, foods rich in vitamin C, such as citrus fruits, cabbage, Brussels sprouts, strawberries, kiwifruit, green peppers, and grapefruit, should be included in the diet [191]. Because of its potent reducing and “antioxidant” qualities, this vitamin aids in preventing radicals from oxidizing cells, “cell membranes”, and “macromolecules”, including “DNA” as well as proteins [192].

“Vitamin E” is an aqueous compound that binds to the membrane and possesses strong antioxidant properties, in addition to having the capacity to scavenge free radicals [190]. Grain, sunflower seeds, peanuts, rapeseed, vegetables, almonds, maize, soybeans, and meats are all good sources of specific “nonenzymatic antioxidants” [193]. Due to vitamin E’s effectiveness in halting lipid peroxidation and collagen fiber cross-connection, ingestion of the nutrient helps reduce signs of aging skin [5]. Vitamin E has been shown to relieve sun rays along with UV rays-related skin harm [194].

The benefits of C and E vitamins are complementary. For instance, when ultraviolet-induced compounds react within cells, the chain reaction of lipid oxidation begins with membranes containing PUFA. During this step, the antioxidant “d-α-tocopherol” transforms into a “tocopheroxyl radical”, which then renews itself with the help of ascorbic acid [179]. Several food products contain tocopherol, like “corn”, “seeds”, “vegetable oils”, and “soy” [5]. Organic versions of “vitamin E” also protect the skin from “collagen cross-linking” and “lipid peroxidation”, two conditions linked to aging “skin. Comparative research has shown that oral administration of both the E and C vitamins can improve the light-protective potency when compared to “monotherapies” [195]. In multiple experiments, a control group of 100–180 mg of vitamin C per day was given to approximately 33 participants over a period of four weeks. The study’s findings revealed that oral supplementation of “vitamin C” increased the skin’s ability to remove “free radicals” by 22% (for 100 mg) and 37% (for 180 mg) above baseline levels [101].

### 3.7. PUFAs or “Polyunsaturated Fatty Acids”

The main type of fat that is essential for preventing age-related illnesses is polyunsaturated fats (PUFAs). Prostaglandins (a precursor to PUFAs) are crucial for controlling cholesterol levels [196,197,198]. The upcoming paragraphs detail their newly discovered involvement in aging.

#### 3.7.1. Omega-3 Polyunsaturated Fatty Acid

In addition to preventing senile dementia [199], omega-3 PUFA helps control hypertension and platelet aggregation [197]. Human intestinal flora is thought to have a role in mediating the anti-aging effects of “omega-3 fatty acids”, according to Xie et al. [200]. “Omega-3 polyunsaturated fatty acids” are mostly found in “walnuts”, “pumpkin seeds”, “sunflower seeds”, and “Calanus oils” [199,201,202]. The primary ways that “polyunsaturated fatty acids (PUFAs)” work is by competing with the generation of “arachidonic” and “eicosanoid acids” to reduce “inflammation”. Medical as well as laboratory studies have demonstrated the significance of micronutrients, including specific microelements, for internal and skin health [203]. According to studies conducted on humans and experiments, omega-3 PUFA has pro-cognitive and neuroprotective effects on aging brains. Additionally, it has been demonstrated that peripheral intensities of omega-3 PUFA and regional gray matter (GM) volume are positively correlated and that “cognitive” impairments and “dietary omega-3 PUFA” quantities are negatively correlated [204,205]. These findings suggested that dietary omega-3 polyunsaturated fat intake might enhance front-hippocampal GM organization and function during normal aging.

Furthermore, studies have shown that German women in their middle years (40–60 years old) who have low levels of omega-3 PUFAs are more likely to experience cardiovascular problems [206]. Research has shown that “omega-3 polyunsaturated fatty acids” enhance dendritic synaptic spines, stimulate hippocampal neurogenesis in old age, and raise levels of many signaling molecules that support flexibility. Senior people with “omega-3 polyunsaturated fatty acids” possess positive “anti-inflammatory” benefits linked to improved cognitive performance, indicating the substance’s efficacy in preventing integrity loss and the loss of white and gray matter volume [207,208]. Recent research has suggested that plasma homocysteine levels may have an impact on a relationship among “omega-3-PUFA” as well as “cognitive” decrease in senior people [209]. Various studies have found a connection among “cognitive” issues and “omega-3-PUFA” [210,211].

#### 3.7.2. Omega-6 Polyunsaturated Fat

Omega-3 and omega-6 fatty acids, vital constituents of plasma membranes, also function as building blocks for various bodily molecules, including those that regulate blood pressure and inflammation. Most of the lipids required by humans are synthesized, except for two, namely, omega-3 linolenic acid (ALA) and omega-6 linoleic acid (LA). They are called “essential fatty acids” and need to be consumed through food. These two fats are required in order to both develop and repair, but they are also used to synthesize other “fatty acids”. Docosahexaenoic acid (DHA) and EPA (eicosapentaenoic acid) are essential omega-3 fatty acids essential for overall health and well-being, for example, ALA may be converted; however, due to the less utilization, obtaining these sources through meals is also advised. Both “ALA” and “LA” were fatty acids found in vegetables as well as seed oils. Even though LA amounts are frequently far more than those of ALA, “canola” and “walnut oils” were good providers of ALA.

According to recent studies on *C. elegans*, the “anti-aging” properties of “omega-6-PUFA” have been connected to “autophagy” and, as a result, to a “phenotype” that is resistant to starvation [212,213]. Omega-6-PUFA’s advantageous effects are explained by linoleic acid’s plasma circulation [214]. This presents the basic idea that a proper ω3/ω6 ratio needs to be maintained to minimize any potential negative consequences from consuming an excess of omega-6 PUFAs [215,216].

### 3.8. Additional Organic Ingredients from Herbs

Plants are the source of numerous “phenolic” and “polyphenolic” compounds. The most common type of “flavonoids” (“isoflavones”, “lignans”, “flavones”, and so on) operate as “phytoestrogens” or chemicals with “hormonal-like” properties, usually acting as “estrogen” receptors. Not every one of these chemicals binds to an estrogen receptor to work. These chemicals have been the subject of research demonstrating their effectiveness in lowering cancer and bone loss and their specific targeting to the mammary tissues, colon, and prostate.

Among the many anti-aging effects of veggies is that eating them raw may greatly slow down the course of “Alzheimers disorder” [217]. The majority of them belong to members of various botanical families, including Cucurbitaceae (pumpkin and cucumber), Brassicaceae (radish, broccoli, and red cabbage), Asteraceae (artichoke), Dioscoreaceae (yam), Amaranthaceae (Chinese spinach), Fabaceae (red bean and soybean), etc.

*Allium sativum*, “*Crataegus* spp.”, Seaweeds, “*Ginkgo biloba*”, *Cynara scolymus*, “*Hippophae rhamnoides*”, *Schizandra chinensis*, “*Panax ginseng*”, *Aloe vera*, and *Silibum marianum* were a few examples of medicinal plant sources that are beneficial in preventing age-related illnesses [218,219,220]. As an example, coenzyme Q10 and *Allium sativum* extract applied together had a positive impact on inflammatory markers and the advancement of atherosclerosis [221]. An organosulfur compound from old garlic called S-Allylcysteine can slow down aging by controlling mitochondrial processes [222]. On the livers of aged mice, “*Silybum marianum*” seed oil significantly reduced “oxidative damage” and enhanced mitochondrial metabolism [223]. Extracts from *Ginkgo biloba* have been used for a long time to treat various degenerative disorders, such as memory loss, cerebrovascular disorders, and aging skin, due to their potential to prevent apoptosis and mitochondrial malfunctions [105].

Nutraceuticals, wholesome foods, and dietary additives are all made with a variety of compounds that have been shown to improve health [224]. Nutritious diets are attracting attention worldwide because of their potential to reduce the appearance of skin age signs and symptoms (Table 2) [225]. It is interesting to note that fruits, being high in “carotenoids”, phenolic compounds, and antioxidant potential, are a vital supply of natural phytochemicals that help minimize the appearance and symptoms of wrinkles on the skin [226].

**Figure 2:**
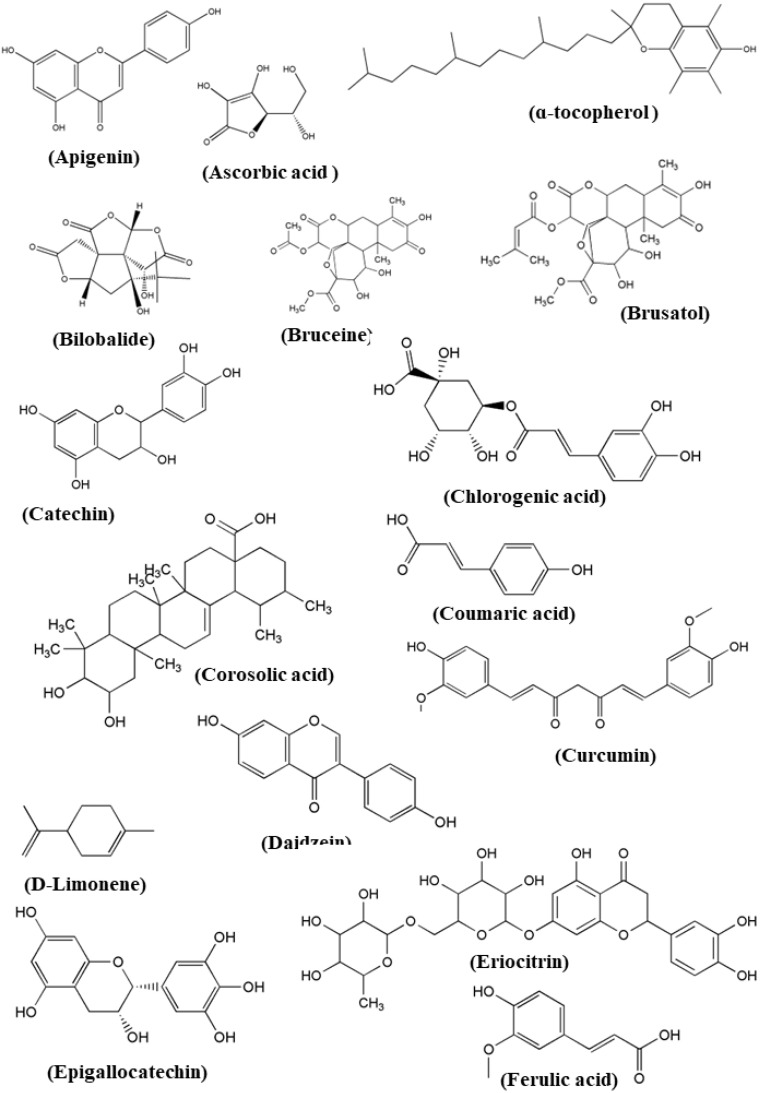

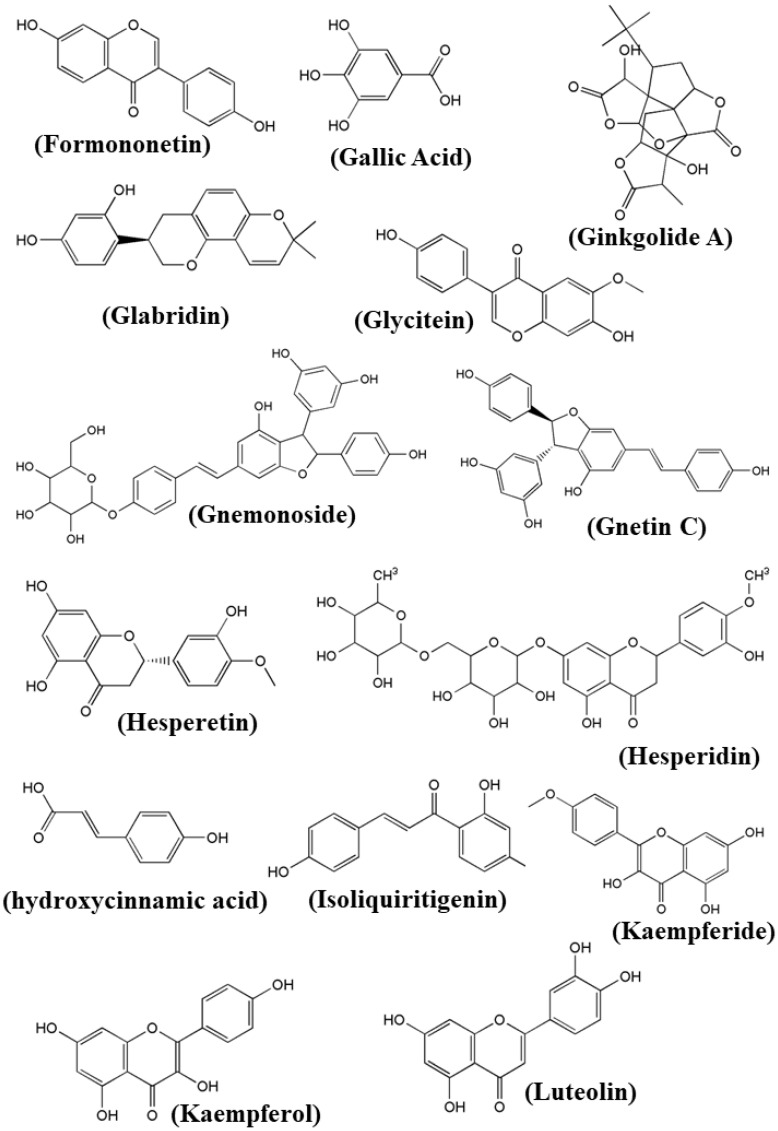

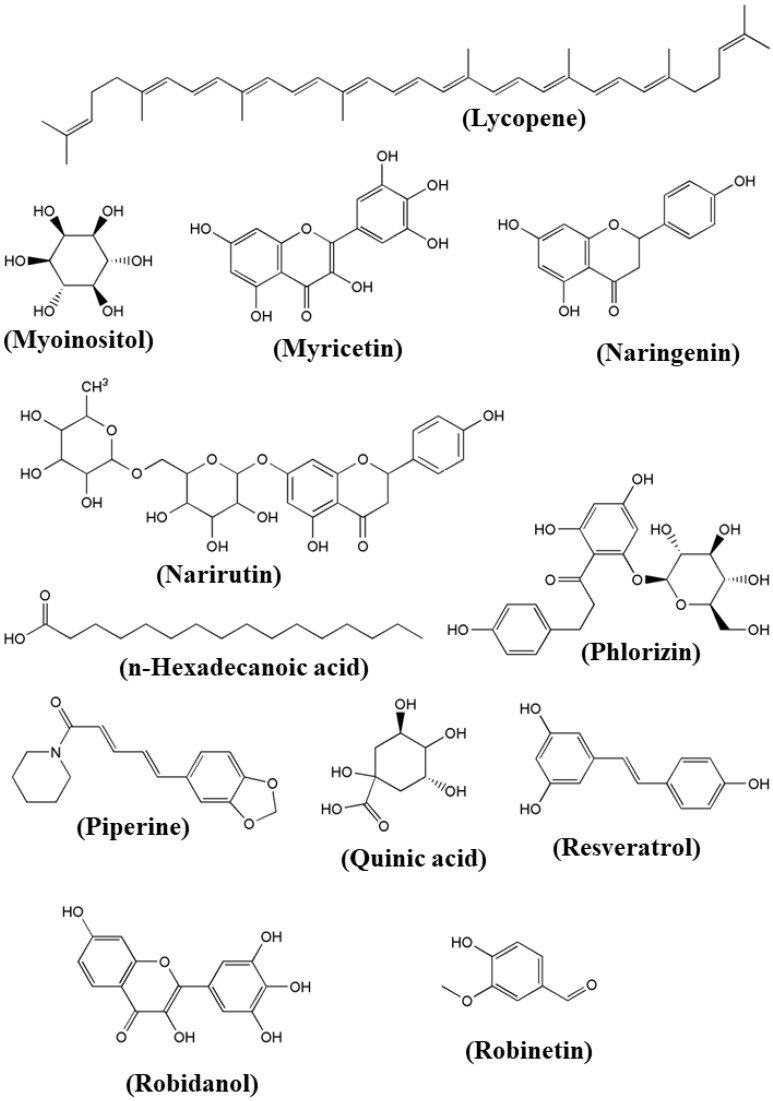
Some important chemical compounds having anti-aging properties.

Various chemical compounds present in the plant that is used in traditional foods have different mechanisms as anti-aging properties. In Table 3, some of the important phytocompounds with their modes of action are listed (Figure 2).

## 4. Molecular Processes Behind the Actions of Plant-Based Foods and Dietary Additives and Mechanism of Anti-Aging Effects

A gradual and cumulative decline in physical health and functioning is a hallmark of the complex, multifaceted, and unavoidable process that is human aging [259]. It acts as a well-known significant risk factor that raises a person’s vulnerability to illnesses, particularly in older persons. Nutrient-sensing mechanisms are the main cellular processes by which natural goods prolong life expectancy. The most researched pathways associated with longevity include AMPK, SIRT/NAD^+^, IIS, mTOR, and p53. One metabolic organelle that interacts with several metabolic signaling mediators is the mitochondria. Over time, previously mentioned lifespan mediators’ effects on mitochondrial activity and metabolic signaling usually deteriorate. In the elderly, this breakdown throws off metabolic health, which can result in cardiac and metabolic problems [260]. A tumor may be a complicated illness with “non-specific age-occurrence” aspects, according to Torre et al. [261]. Still, there are several “hallmarks” that are somewhat overlapping in both cancer and aging. The relationship, if any, between cancer-related changes in cell metabolism and aging-related dysregulation of nutrition sensing is complicated. The IIS cycle, for instance, is involved in lifespan; nevertheless, malignant cells hijack this system to promote unchecked growth. Telomere erosion and stem cell depletion are two further aging characteristics that are expected to inhibit tumor formation [262]. The IIS signaling process was the first identified lifespan-regulating signaling system and is crucial for the regulation of aging [263]. “IGF-1” activates complicated internal signaling pathways by phosphorylating glucose receptor substrates and starting the “PI3K-Akt” as well as “p38/MAPK signaling” pathways through binding with “high-affinity IGF-1 receptors” in the cellular surface. Numerous gene “transcription factors” become restricted in their expression when the “IIS pathway” is activated, including “DAF-16/Forkhead box O (FOXO)”, “SKN-1/NRF2”, and thermal “shock factor-1”. Results on longevity are increased by this inhibition of descending gene targets [264]. “Yuan et al.” have demonstrated that the two fractions of “*Rehmannia glutinosa*” polysaccharide reduce the generation of lipofuscin in Caenorhabditis worms, absorb extra ROS, and improve SOD and CAT activities. Through the IIS system, the astragalus neutral polysaccharides could additionally promote daf-16′s nucleus positioning and increase *Caenorhabditis elegans’* lifetime [265].

As genes, proteins such as “p53” and “FOXO” control a variety of signaling pathways that govern “metabolism”, “programmed cell death”, and the “cell cycle”. A downstream target coding protein known as p21 is an inhibitor of “cyclin-dependent” kinase “protein inhibitor” when p53 expression is elevated, hence initiating cellular aging processes. Senescence of the cell may result from p21 protein stimulation, which can prevent cells from moving from the phase of G1 into the S phase [266]. Apart from the observed inverse alteration of the activity with “p53” and “p21” molecules in the coronary tissue of aging mice, Yu et al. [267] revealed significant improvements in age-associated symptoms among animals following a 10-month ginsenoside Rb1 intervention. According to Yu et al. [267], the intervention improved metabolic disorders and affected “apoptosis” and cell cycle advancement.

The enzymatic (“α1, α2, β1, and β2”) and regulating (“γ1, γ2, and γ3”) components make up the heterotrimeric complex known as AMPK. Various combinations of α, β, and γ can be formed by different isomers that are expressed in various organs connected to metabolism. Cellular metabolism of energy depends on AMPK in significant ways. By stimulating and phosphorylating many downstream target molecules in response to a boost within the intracellular AMP/ATP ratio, AMPK decreases ATP utilization while increasing ATP synthesis. Catabolism is the final result of these biological activities. Cellular anabolism is facilitated by AMPK inhibition, which is brought on by a drop in the AMP/ATP ratio [268]. Age-related declines in AMPK activity are being associated with age-related illnesses, and AMPK activity decline could be one of these factors. In addition, “AMPK” controls animal longevity, physical rhythm, and the establishment and maintenance of cell division, all of which are essential processes involved in cell expansion and division. Research has indicated that age-related disorders are largely caused by energy imbalances and a loss in AMPK activity [269,270,271]. According to Xu et al. [272], BBR and *Rhizoma coptis* are promising anti-aging drugs that extend the lifetime of many species and reduce age-related disorders by promoting healthy aging through AMPK activation and antioxidative qualities.

A unique “serine/threonine protein kinase”, “mTOR” belongs to the “PI3K-associated kinase” family. It engages in several protein interactions to generate a pair of unique groups known as “mTORC1” and “mTORC2”. Numerous biological processes, including autophagy, proliferation, survival, and “cell cycle” regulation, are regulated by these “complexes”. Growth factors are known to promote mTORC1, but energy stress, AMPK activity, acid shortage, hypoxia, endoplasmic reticulum stress, and genotoxic stress all block it. Nutritional deficits have little effect on mTORC2 [273]. In many different taxa, including yeasts, worms, flies, and humans, mTOR signaling is intimately linked to the aging processes [274,275]. Autophagy is also significantly inhibited by mTOR, and autophagy is encouraged when the mTOR signaling pathway is inhibited. In addition to the aging process itself, increased autophagy has been found to be associated with several age-related conditions, including neurological disorders, metabolic diseases, and cancer [276].

The molecular mechanisms behind the anti-aging effects of phytocompounds, which are derived from plants, are diverse and complex. There is a growing body of research suggesting that various plant-based chemical compounds possess anti-aging properties, which can be attributed to their ability to modulate several cellular pathways and processes associated with aging. The anti-aging effects of natural products stem from their ability to mitigate oxidative stress, a process intricately linked to aging [277]. Research dating back to 1956, with Harman’s free radical theory of aging, established the notion that excessive reactive oxygen species (ROSs) and oxidative stress resulted in progressive macromolecular damage [278,279]. This oxidative stress primarily originates from intracellular sources such as mitochondria, peroxisomes, and cytosolic biocatalysts; among them is NADPH oxidase. Mitochondria, particularly through the electron transport chain (ETC), are identified as major ROS generators [280,281]. Cells employ antioxidant defense processes to counteract oxidative stress. These mechanisms involve nonenzymatic antioxidants such as “glutathione” and “vitamins” and enzymatic systems comprising “catalase”, “superoxide dismutase” (“SOD”), and “glutathione peroxidase” (“GPx”) [282,283,284]. Studies in model organisms like *C. elegans* and mice have shown that genetic alterations affecting mitochondrial function and ROS levels can influence lifespan, suggesting a direct association between aging and oxidative stress [279]. The detrimental effects of oxidative stress extend beyond mitochondria to include damage to both mitochondrial DNA (mtDNA) and nuclear DNA [285]. “ROS-induced damage” to mtDNA contributes to “mitochondrial” dysfunction, which exacerbates oxidative stress and impairs cellular energy production. Moreover, oxidative damage to nuclear DNA triggers cellular responses like senescence, characterized by irreversible growth arrest [286,287]. Additionally, protein oxidation and aggregation, caused by ROS, contribute to cellular senescence and aging pathology. Natural products rich in antioxidants offer a promising avenue for combating oxidative stress and its associated aging effects [288,289,290]. By bolstering antioxidant defenses and targeting pathways involved in mitochondrial function and DNA damage repair, these compounds exert protective effects against age-related decline. Through their multifaceted mechanisms, natural products hold potential as therapeutic interventions for age-related ailments and stimulating healthy aging [291].

**Figure 3:**
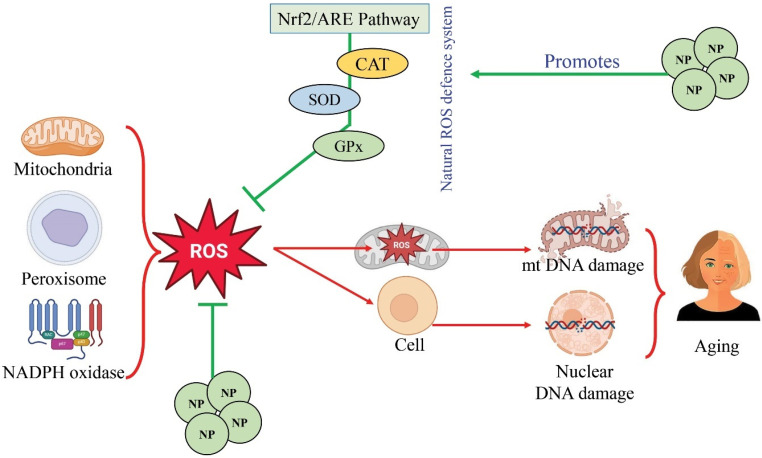
Mode of action of chemical compounds from plants in anti-aging. Here, NP denotes ‘natural products’.

Natural compounds exert their anti-aging effects by targeting the Nrf2/ARE pathway, a crucial cellular defense mechanism against oxidative stress [292,293]. Reactive oxygen species (ROSs) are not continuously created by skin cells in the presence of antioxidants such as polyphenols and ascorbic acid, along with endogenous antioxidant enzymes comprising “catalase” (Cat), “glutathione peroxidase” (GPx), and “superoxide dismutase” (SOD) [279]. These antioxidants neutralize free radicals, preventing oxidative damage to cellular components. Exposure to ultraviolet A and ultraviolet B radiation increases reactive oxygen species (ROSs) production beyond the capacity of enzymatic antioxidants, resulting in the loss of oxidative stress and endogenous antioxidants [294]. In response, transcription factors like NRF2, which stands for nuclear factor erythroid 2, are activated to regulate cytoprotective genes, mitigating oxidative stress [295]. Nrf2, normally inactive in the cytosol due to interlinkage with “Kelch like-ECH-associated protein 1” (“Keap1”), is activated during oxidative stress. This activation can occur through phosphorylation by kinases or oxidative modification of Keap1, causing Nrf2 to be released from Keap1. After being activated, Nrf2 enters the nucleus of the cell, where it binds to “antioxidant response elements” (ARE) in the promoters of target genes and forms heterodimers with sMaf proteins [236,294]. The genes glutathione synthetase and heme oxygenase 1 (HO-1) are responsible for the protection of cell responses and can be genetically regulated by ARE, a cis-acting regulatory sequence. The upregulation of these enzymes helps combat oxidative damage and maintain cellular homeostasis. Natural compounds, through their ability to modulate the Nrf2/ARE pathway, enhance the expression of cytoprotective genes and bolster cellular defenses against oxidative stress. By promoting Nrf2 activation and subsequent transcription of ARE-regulated genes, these compounds mitigate oxidative damage, thereby preventing premature aging and photoaging of the skin. Thus, targeting the Nrf2/ARE pathway represents a promising approach for anti-aging interventions using natural compounds [294,296]. In Figure 3, the mode of action of chemical compounds (natural products), denoted by NP against aging, is illustrated.

## 5. Pre-Clinical and Clinical Studies on Anti-Aging Effects of Traditional Plant-Based Foods

Many clinical trials exploring the enhancement of skin health through chemical compounds derived from traditional edible plants primarily focused on developing promising nutricosmetics. A clinical investigation was conducted by researchers to explore the photoprotective benefits of consuming flavanols found in cocoa products. They found that a cocoa drink containing flavanols showed some improvement in reducing facial wrinkles and enhancing skin elasticity. This indicates that consuming cocoa beverages could serve as a preventive measure against photoaging. Nevertheless, additional investigation is necessary to validate the beneficial effects of flavonoids on skin well-being [297]. In another aspect, Passiflora seeds contain high levels of piceatannol, a powerful phenolic compound known for its antioxidant properties, which can effectively mitigate and prevent damage caused by ultraviolet radiation (UVR) on the skin. Passion fruit can be a beneficial dietary supplement due to its beneficial properties. An experiment on passiflora fruit extract pills reduced the skin’s water loss after two months of consuming a solution containing piceatannol [298]. Some results of the clinical trials are tabulated in Table 4.

## 6. Conclusions

Environmental factors as well as genetics have a role in the complex and ever-expanding biological process of aging. Nowadays, aging has been associated with consuming a diet that is imbalanced and deficient in many essential nutrients. Nutraceuticals are becoming more and more valued and are thought to be essential for extending life and supplying molecules rich in antioxidants. Concerning delaying or even stopping the aging process, nutraceuticals as dietary supplements consequently offer considerable interest. Supplemental foods are incorporated into diets for long-term health advantages due to their associated benefits. This review underscores the significant anti-aging potential of traditional plant-based foods, driven by a rich array of bioactive composites such as polyphenols, flavonoids, and phytochemicals. These natural ingredients exhibit diverse mechanisms of action, comprising antioxidant, anti-inflammatory, and cell-regenerative properties, which collectively contribute to their ability to combat the aging process. Moving forward, future research endeavors should focus on elucidating the precise molecular mechanisms underlying these effects, conducting larger-scale clinical trials to validate their efficacy and safety in humans, and exploring synergistic interactions between different plant-derived compounds. Additionally, the exploration of traditional plant-based foods from various cultural backgrounds may reveal novel bioactive compounds with unique anti-aging properties, ultimately paving the way for the development of innovative strategies to promote healthy aging and longevity.

## Figures and Tables

**Table 1:** A list of plant biomolecules used as adaptogens.

Phytoconstituent	Biological Effect	Tests/Models	Mechanism of Action	Ref.
Curcumin	Adaptogenic activity Antidepressant-like effects	Chronic stress, Chronic unpredictable stress and forced swimming	Alter functional homeostasis and memory deficit and cause normalization of the hyper-activated HPA axis with subsequent decrease in corticosterone secretion. Acts on the serotonergic system, which may be mediated by an interaction with 5-HT1A/1B and 5-HT2C receptors responsible for depression.	[22,24]
Rutin	Anti-stress activity Anxiolytic-like activity	Forced swimming, Tail suspension, Elevated plus-maze Elevated plus-maze, Ambulatory activity	Modulates GABA receptors and also acts as an NMDA receptor antagonist. Cause inhibition of prostaglandin synthesis, thereby regulating HPA axis activity under basal and stress conditions. Modulates GABAergic neurotransmission in the basolateral amygdala.	[25,26]
Ginsenosides Rb1 and Rg1	Adaptogenic activity	Radical scavenging, Nrf2 activation, Mitochondrial dysfunction, BBB permeability	Attenuates oxidative stress and mitochondrial dysfunction, thus resulting in reduced apoptotic cell death. The promising effects are due to the activation of cytoprotective Nrf2 signaling and a mitochondrial-targeted protective action.	[27]
Glycyrrhizin	Antidepressant-like activity and anti-stress activity	Forced swimming, Tail suspension, Immobilization stresses locomotor activity, Muscle co-ordination	Acts by interaction with α1-adrenergic and D2-receptors, thereby increasing the levels of norepinephrine and dopamine in brains, and also has MAO-inhibiting activity. Attenuates the HPA axis activation and free radical scavenging activity.	[28,29]
Piperine	Antidepressant-like activity and anti-anxiety-like activity	Elevated plus-maze, Light and dark box, Social interaction, Tail suspension, Forced swimming, Open field test.	Possibly mediated through the benzodiazepine-GABA receptor and an increase in GABA levels and inhibition of neuronal nitric oxide synthase. Cause augmentation of the neurotransmitter synthesis or the reduction in the neurotransmitter reuptake and also mediated via the regulation of the serotonergic system.	[30,31]
Eugenol	Anti-stress activity	Restraint stress model	Activity is mediated through the HPA axis and BMS pathways and also changes in brain noradrenergic, serotonergic, and dopaminergic systems in the hippocampus, hypothalamus, prefrontal cortex, and amygdala.	[32]
Andrographolide	Adaptogenic activity	Stress-induced hyperthermia, Pentobarbital-induced hypnosis	Cause upregulation of the benzodiazepine site of GABA-A receptors, which are involved in stress-triggered physiological responses.	[33]
Ocimumosides	Anti-stress activity	Restraint stress model	Anti-stress activity by normalizing acute stress-induced hyperglycemia, corticosterone levels, creatine kinase, and adrenal hypertrophy.	[34]
Astragaloside IV	Anti-stress activity	Immobilized stressors, Elevated plus-maze	Astragaloside IV may ameliorate anxiety via serotonergic receptors and inflammation by decreasing serum levels of corticosterone, IL-6, and TNF-α.	[35]
Astragaloside II	Immunomodulatory activity	Splenic T-lymphocyte activation, Cyclophosphamide-induced immunosuppression	Astragaloside II enhances T cell activation by regulating the activity of CD45 PTPase in primary T cells. Astragaloside II also increased IL-2 and IFN-γ secretion, upregulated IFN-γ and T-bet in primary splenocytes, and promoted primary CD4 + T cells.	[36]
Bergenin	Immunomodulatory activity	Adjuvant-induced arthritis	Inhibits IL-2 production by CD4 + T-cells, which are regulators of immune response, stimulates the synthesis of IFN-ϒ in T-cells, and also induces the secretion of pro-inflammatory cytokines such as TNF-α by activated macrophages. The inhibition of IL-2 is possibly responsible for reduced IFN-ϒ secretion by CD8 + T cells and TNF-α by macrophages and also promotes IL-4 and IL-5 production.	[37]
Syringin	Immunomodulatory activity	Croton oil and arachidonic acid-induced mouse ear edema	Inhibits TNF-α production and CTLL-2 cell proliferation and thus may possess anti-allergic activity.	[38]
Berberine	Antidepressant and anti-anxiety-like activity	Forced swimming, Elevated plus-maze, Locomotor activity	Acts by modulating hypothalamic corticotrophin-releasing factor and the noradrenergic system in the CNS and decreasing serotonergic system activity by activating somatodendritic 5-HT1A and inhibiting postsynaptic 5-HT1A and 5-HT2 receptors.	[39,40]
β-pinene	Antidepressant-like activity	Forced swimming	Acts through interaction with the serotonergic pathway through postsynaptic 5-HT1A receptors and also interacts with the adrenergic system through β-receptors and the dopaminergic systems through D1 receptors.	[41]
Zeatin	Immunomodulatory activity	Thioglycollate-induced peritonitis	Modulates T lymphocyte activity via the adenosine A2A receptor, which induces the production of cAMP, potently inhibits the production of both TH1 and TH2 cytokines, and also modulates, either directly or indirectly, both humoral and cell-mediated responses.	[42]
Scopoletin	Antidepressant-like activity	Forced swimming, Tail suspension, Open field test	Interact with the 5-HT2A/2C, α1, and α2, D1, and D2 receptor systems, thereby producing an antidepressant-like effect.	[43]
Ginkgolide B	Adaptogenic and anti-stress activity	In situ hybridization of CRH and AVP mRNA	Acts at the hypothalamic level, modulating the monoaminergic inputs to the CRH-synthesizing cell bodies depending upon both the nature of stress and substance.	[44]
Quercetin	Anti-fatigue activity	Forced swimming	Improves endurance capability to fatigue during exhaustion and also prevents endothelial dysfunction via enhancing the activities of antioxidant enzymes and attenuating the levels of inflammatory cytokines.	[45]
Gallic acid	Antidepressant-like activity	Sucrose preference test and forced swimming	Inhibits MAO-A activity and increases levels of monoamine in the brain, reduces plasma nitrite levels, and reduces nitrosative stress, thus playing a key role in chronic stress-induced depression.	[46]
Valerenic acid	Anti-stress activity Anxiolytic activity	Forced swimming and elevated plus-maze	Mitigate stress by decreasing the turnover of 5-HT to 5-hydroxyindoleacetic acid and NE to 3-methoxy-4-hydroxyphenylethyleneglycol sulfate in the hippocampus and amygdala. Modulates the GABA-A channel and possesses anxiolytic activity.	[47,48]
Picracin	Immunomodulatory activity	Pro-inflammatory cytokine release, DTH response	Inhibits mitogen-induced proliferation of T cells and is a potent inhibitor of IL-2 release through induction of apoptosis.	[49]
Esculetin	Anti-inflammatory and depressive-like activity	Tail suspension, Forced swimming, Open field test	Inhibits pro-inflammatory cytokines, including interleukin-6, interleukin-1β, and tumor necrosis factor-α, and also attenuates inducible nitric oxide synthase and cyclooxygenase-2 protein expression by inhibiting the nuclear factor-κB pathway in the hippocampus, causing upregulations of brain-derived neurotrophic factor and phosphorylated tyrosine kinase B protein expression in the hippocampus, which provides neuroprotection.	[50]
Catechin	Anxiolytic activity	Forced swimming, Elevated plus-maze	Inhibits the HPA axis-associated psychological dysfunction induced by corticosterone and modulates hypothalamic CRF activity and the noradrenergic system within the CNS.	[51]
Asiaticoside	Antidepressant-like activity	Tail suspension, Forced swimming	Regulates the α2-adrenergic receptor and increases the level of adrenaline in the brain.	[52]
Tumerone	Antidepressant-like activity	Forced swimming, Tail suspension, Open field test	Increase the level of 5-HT in the cortex, striatum, hippocampus, and hypothalamus; the level of NE in the striatum and hippocampus; the level of 3-methoxy-4-hydroxyphenylglycol and 3,4-dihydroxyphenylacetic acid in the hypothalamus; the level of 5-hydroxyindoleacetic acid in the striatum; and the level of DA in the striatum, hippocampus, and hypothalamus.	[53]
Rosavin	Adaptogenic activity	Forced swimming light/dark test, Tail-flick latencies	Modulates biogenic monoamines in the cerebral cortex, brain stem, and hypothalamus. In the cerebral cortex and brain stem, the levels of norepinephrine and dopamine decreased, while that of serotonin increased due to changes in monoamine levels, that is, the inhibition of monoamine oxidase and catechol-o-methyltransferase.	[54]
Shatavarin	Immunomodulatory activity	Human peripheral blood lymphocyte stimulation assay	Stimulates immune cell proliferation, induces IgG and interleukin-12, and inhibits IL-6 production. It also had strong modulatory effects on the Th1/Th2 cytokine profile.	[55]
Salidroside	Anti-fatigue activity	Forced swimming	Decrease the activities of CK and CK-MB and increase the GSH-Px and SOD activities, and also decrease the MDA content in liver tissue.	[56]
β-sitosterol	Anxiolytic-sedative activity	Pentobarbital-induced sleeping time	Modulates the GABAA receptor and produces an anxiolytic effect similar to that of benzodiazepines.	[57]
Ellagic Acid	Antidepressant and anti-anxiety activity	Novelty-suppressed feeding, Forced swimming, Sucrose intake test	Acts probably by interaction through adrenergic and serotonergic systems and causes inhibition of inducible NOS, thereby acting as an antidepressant.	[58]
Puerarin	Antidepressant and anti-stress activity	Tail suspension, Forced swimming	Ameliorates depression and pain via activating ERK, CREB, and BDNF pathways, inhibits corticotropin-releasing hormone, corticosterone, and adrenocorticotropic hormone, and normalizes the activity of the serotonergic system, thereby preventing HPA axis dysfunction.	[59,60]
Chlorogenic acid	Antidepressant activity	Tail suspension, Elevated plus-maze.	Crosses the blood-cerebrospinal barrier, exhibits neuroprotection, and promotes serotonin release through enhanced synapsin I expression.	[61]
Ursolic acid	Antidepressant and anxiolytic-like activity	Tail suspension, Forced swimming, Open field test	Action is mediated by an interaction with the dopaminergic system through the activation of dopamine D1 and D2 receptors.	[62]
Caffiec acid	Antidepressant and anxiolytic-like activity	Elevated plus-maze, Open field test	Indirectly modulates α1-adrenoceptors, that is, α1A-adrenoceptors in cortical membranes, and directly modulates second messengers acting through glutamate or GABAergic receptors, thereby being involved in the expression of its antidepressive and/or anxiolytic-like effects.	[63,64]
Sulforaphane	Antidepressant and anxiolytic-like activity	Forced swimming, Tail suspension, Chronic stress, Open field test	Normalize the stress-induced HPA axis dysfunction and have inhibitory effects on the inflammatory response to stress.	[65]
Chicoric acid	Antidepressant activity and immunomodulatory activity	Forced swimming, Learned helplessness Chronic restraint stress-induced altered T lymphocyte distribution	Modulates nor-adrenaline, dopamine, and 5-hydroxy tryptamine in chronically stressed conditions. Imparted immune stimulation by upregulating the expression of CD28 and CD80 and downregulating CTLA-4; stimulatory effect on IL-12, IFN-gamma, and IL-2; and suppression of the increased IL-10 and also lower corticosterone levels, thereby showing its normalizing effect on the HPA axis.	[66,67]
3,4,5-trimethoxycinnamic acid	Anti-stress, anxiety, and depression-like activity	Repeated cold exposure test	Augment norepinephrine in the brain and also ameliorate chronic stress and induce ΔFosB protein and SC1 mRNA expression in a nucleus accumbens shell subregion.	[68,69]
Rosmarinic acid	Anxiolytic-like activity	Elevated plus-maze, Step-down avoidance, Open field test	Involved in direct modulation of a second messenger through glutamate receptors, since these are directly involved in several CNS disorders, and the anxiolytic effect is seen at lower doses without affecting the short- and long-term memory retention or locomotion, exploration, and motivation.	[70]
Ferulic Acid	Immunomodulatory activity	Carbon clearance, Neutrophil adhesion, Serum immunoglobulins, DTH response	Acts by stimulating cell-mediated immunity as well as humoral immunity by acting on B-cells and T-cells.	[71]

**Table 2:** List of numerous plants and their constituents with anti-aging properties.

Scientific Name	Common Name	Bioactive Compounds	Bioactivities	References
*Carthamus tinctorius*	Safflower seed oil	Phenol	Collagenase assay inhibition and suppression in the test for elastase	[227]
*Chaenomeles sinensis*	Chinese quince	β-1,4-xyloglucan	Inhibition of cutaneous extracellular matrix proteases’ activity: collagenase and elastase	[228]
*Citrus unshiu* Marcov; “*Citrus sunki*” Hort. Ex Tanaka; “*Citrus reticulate*” Blanco”; “*Vitis vinifera* L.”; *Citrus sinensis* Osbeck	Mandarin, grapes	Ascorbic acid, narirutin, hesperidin	A hairless mouse model exposed to UV radiation showed increased collagen levels, reduced skin layer thickness and wrinkling, and higher levels of enzymes fighting free radicals.	[229]
“*Citrus reticulate*” Blanco	Mandarin orange	D-limonene and n-hexadecane.	Suppression of “elastase”, “collagenase”, and anti-enzyme activity	[230]
“*Cucumis sativus* L.”	Cucumber	Ascorbate ascorbic acid	In vitro suppression of MMP-1, elastase, and hyaluronidase	[231]
“*Citrus sinensis* L.”	Sweet orange	Flavanones, anthocyanins, ascorbic acid, and hydroxycinnamic acid	Translocation of “NF-B and AP-1” along with “cleavage of procaspase-3”	[232]
“*Curcuma longa*”	Turmeric	Curcumin	Decrease in the anti-aging inflammatory marker C-reactive protein (CRP) levels	[233]
“*Citrus limon*”	Lemon	Eriocitrin (polyphenols)	Delay in locomotor atrophy and raised aging-related scores	[234]
“*Camellia sinensis* L.”	Green tea	EGCG (epigallocatechin-3-gallate)	Prolongation of life with reactive oxygen species (ROSs)	[235]
“*Camellia sinensis* L.”	Orange Pekoe black tea	Epigallocatechin gallate	Elastase activity inhibition	[236]
“*Daucus carota* L.”	“Carrot”	Carrot glycoprotein	Defense of “cell membranes” and neutralization of “reactive oxygen”	[237]
“*Emblica officinalis* L.”	Indian gooseberry	Elaeocarpusin, gallic acid, ascorbic acid	“Procollagen 1” protected against “UVB-induced depletion” by inhibiting “UVB-induced collagenase-1”, “inhibited collagenase”, raising “tissue inhibitor of metalloproteinases level”, and induced cell cycle	[238]
*Emblica officinalis* L.	Indian gooseberry	Vitamin C	Increased endopeptidase content and decreased levels of cutaneous fibroblast matrixins.	[239]
“*Ginkgo biloba* L.”	Maidenhair tree	Isorhamnetin-3-O-glucoside, kaempferol 3-O-β-D-glucopyranoside, myricetin, bilobalide, ginkgolide A	Degradation of “oxidative stress (ROSs) and human interstitial collagenase (MMP-1) inhibition in “fibroblasts of human skin.	[240]
*Tamarindus indica*; *Nephelium lappaceum* L.; Litchi *chinensis*;	Litchi, rambutan, tamarind	Epigallocatechin, gallic acid, ferulic acid	Tyrosinase and TRP-2 inhibition inhibit the synthesis of “melanin in B16F10 melanoma cells”; collagenase and elastase suppression are effective.	[241]
“*Musa spaientum*”	Banana	Corosolic acid	Impact of inhibition on MMP activity	[242]
“*Momordica charantia* L.”	Bitter gourd	Resveratrol	Increasing antioxidative stress and regulating the yeast genes’ expression like “UTH1”, “SKN7”, “SOD1”, and “SOD2”.	[243]
“*Oryza sativa*”	Rice	Coumaric acid and vanillin	Inhibitory action of elastase	[244]
*Panax ginseng*	Asian ginseng	Gingenoside	Stimulation of Transforming growth factor-beta (TGF-β)” in dermal fibroblast cells” promotes the synthesis of collagen.	[245]
“*Prunus dulcis*”	Almonds	α-tocopherol	In postmenopausal women, wrinkle severity is reduced	[246]
*Sclerocarya birrea*	Marula	Catechin, quinic acid, epicatechin gallate, and epigallocatechin gallate	Indicated actions to inhibit collagenase	[247]
“*S. zalacca* (Gaert.) Voss”	Snake fruit	Coffee tannic acid and 3-caffeoylquinic acid	Collagenase suppression	[248]

**Table 3:** Plant-based chemical compounds and their mode of action to combat the aging process.

Compounds	Present in Plant	Mode of Action	References
Anthocyanins	*Citrus sinensis* L.	Translocation of NF-B and AP-1. Cleavage of procaspase-3	[232]
Apigenin	*Spatholobus littoralis* Hassk.	Elastase activity inhibition	[249]
Ascorbic acid	*Emblica ocinalis* L.; *Cucumis sativus* L.; *Citrus sunki* Hort. Ex Tanaka; *Citrus unshiu* Marcov; *Citrus sinensis* Osbeck; *Citrus reticulata* Blanco; *Vitis vinifera* L.	Inhibited type-I collagen collagenase; Increased TIMP-1 level; Inhibited cellular proliferation; Protected procollagen 1 against UVB-induced depletion; Inhibited UVB-induced MMP-1; Promoted procollagen content; Inhibited matrix metalloproteinase levels in skin fibroblasts; Inhibited hyaluronidase, elastase, and MMP-1	[229,231,238,239]
ɑ-tocopherol	*Prunus dulcis*	Reduced wrinkles in postmenopausal women	[250]
Bilobalide	*Ginkgo biloba* L.	Preventing ROS and MMP-1 breakdown in skin cells.	[251]
Bruceine	*Rhus javanica* L.	Anti-elastase activity	[252]
Brusatol	*Rhus javanica* L.	Potential anti-aging	[252]
Catechin	*Sclerocarya birrea* *Cosmos caudatus* Kunth *Spatholobus littoralis* Hassk.	Collagenase inhibition Antioxidant potential; Inhibition of elastase activity; Prevents skin aging	[227,247,249]
Chlorogenic acid	*Salacca zalacca* (Gaert.) Voss	MMP-1 inhibition; Acts as an antioxidant and anti-inflammatory agent; Prevents aging and toxicity; Having affinity for MMP1, NEP, and PPO3	[248]
Corosolic acid	*Musa spaientum*	MMPs Inhibition	[242]
Coumaric acid	*Oryza sativa*	Elastase inhibition	[244]
Curcumin	*Curcuma longa*	Lower C-reactive protein (CRP) levels, an anti-aging inflammatory marker	[233]
Daidzein	*Spatholobus littoralis* Hassk. *Glycine max* (L.) Merr.	Inhibition of elastase	[249,253]
D-Limonene	*Citrus reticulata* Blanco	Inhibit collagenase and elastase.	[230]
Elaeocarpusin	*Emblica ocinalis* L.	Inhibited type-I collagen collagenase; Increased TIMP-1 level; Inhibited cellular proliferation; Protected procollagen 1 against UVB-induced depletion; Inhibited UVB-induced MMP-1	[239]
Epigallocatechin	*Litchi chinensis*; *Nephelium lappaceum* L.; *Tamarindus indica* *Sclerocarya birrea* *Camellia sinensis* L.	Melanin production suppression; Inhibit elastase and collagenase; Inhibit collagenase; Elastase activity inhibition	[235,236,241,247]
Eriocitrin	*Citrus limon*	Delay in locomotor atrophy	[234]
Ferulic acid	*Litchi chinensis*; *Nephelium lappaceum* L.; *Tamarindus indica*	Melanin production suppression; Inhibit elastase and collagenase; Inhibit collagenase; Elastase activity inhibition	[241]
Formononetin	*Spatholobus littoralis* Hassk.	Prevent skin aging	[249]
Gallic Acid	*Emblica ocinalis* L.; *Litchi chinensis*; *Nephelium lappaceum* L.; *Tamarindus indica*	Inhibited type-I collagen collagenase; Increased TIMP-1 level; Inhibited cellular proliferation; Protected procollagen 1 against UVB-induced depletion; Inhibited UVB-induced MMP-1; Melanin production suppression; Inhibit elastase and collagenase; Inhibit collagenase; Elastase activity inhibition	[229,239]
Gingenoside	*Panax ginseng* Meyer; *Crataegus pinnatifida*	Promotion of collagen synthesis via TGF-activation in human skin fibroblast cells Protective effect against UVB-induced photoaging by regulating procollagen type 1 and MMP-1 expression in NHDFs	[245,254]
Ginkgolide A	*Ginkgo biloba* L.	ROS and MMP-1 inhibition	[251]
Glabridin	*Glycyrrhiza glabra* L.	Tyrosinase and elastase inhibition	[113]
Glycitein	*Spatholobus littoralis* Hassk.	Inhibition of elastase activity	[249]
Gnemonoside	*Gnetum gnemon* L.	Inhibits tyrosinase in the melanogenesis process	[255]
Gnemonoside D	*Gnetum gnemon* L.	Inhibits tyrosinase in the melanogenesis process	[255]
Gnetin C	*Gnetum gnemon* L.	Inhibits tyrosinase in the melanogenesis process	[255]
Hesperetin	*Spatholobus littoralis* Hassk.	Prevent skin aging	[249]
Hesperidin	*Citrus sunki* Hort. Ex Tanaka; *Citrus unshiu* Marcov; *Citrus sinensis* Osbeck; *Citrus reticulata* Blanco; *Vitis vinifera* L.	Increase antioxidant enzyme levels; Decrease skin thickness and wrinkles; Rise collagen levels	[229]
hydroxycinnamic acid	*Citrus sinensis* L.	Translocate NF-κB and AP-1 translocation; Cleaved procaspase-3	[232]
Isoliquiritigenin	*Glycyrrhiza glabra* L.	Inhibition of tyrosinase and elastase	[113]
Kaempferide	*Spatholobus littoralis* Hassk.	Prevent skin aging	[249]
Kaempferol	*Ginkgo biloba* L.	Preventing ROS and MMP-1 breakdown in skin cells.	[251]
Luteolin	*Spatholobus littoralis* Hassk.	Prevent skin aging	[249]
Lycopene	*Cosmos caudatus* Kunth	Antioxidant activity	[249]
Myoinositol	*Cosmos caudatus* Kunth	Antioxidant activity	[227]
Myricetin	*Ginkgo biloba* L.	Preventing ROS and MMP-1 breakdown in skin cells.	[251]
Naringenin	*Spatholobus littoralis* Hassk.	Inhibition of elastase activity	[249]
Narirutin	*Citrus sunki* Hort. Ex Tanaka; *Citrus unshiu* Marcov; *Citrus sinensis* Osbeck; *Citrus reticulata* Blanco; *Vitis vinifera* L.	Increased antioxidant enzyme expression; Reduced skin thickness and wrinkles; Elevated collagen levels	[229]
n-Hexadecanoic acid	*Citrus reticulata* Blanco	Collagenase and elastase inhibition	[230]
Phlorizin	*Eleutherococcus senticosus*	miR135b suppression; Enhances microenvironment; Increases proliferative potential basal epidermal cells	[256]
Piperine	*Piper nigrum* L.	Antioxidant activity	[257]
Quinic acid	*Sclerocarya birrea*	Collagenase inhibition.	[247]
Resveratrol	*Momordica charantia* L.	Antioxidant activity	[243]
Robidanol	*Intsia bijuga* (Colebr.)	Antioxidant activity and antityrosinase enzyme inhibition	[258]
Robinetin	*Intsia bijuga* (Colebr.)	Antioxidant activity and antityrosinase enzyme inhibition	[258]
Vanillin	*Oryza sativa*	Elastase inhibition	[244]
β-1,4-xyloglucan	*Chaenomeles sinensis*	Inhibit dermal proteases: elastase and collagenase.	[228]

**Table 4:** Chemical compounds in clinical trials for anti-aging effects.

Compounds/Extracts Name	Source	Study Outcome	References
Flavanol from cocoa	*Theobroma cacao*	Enhanced the elasticity and reduced wrinkles in the skin.	[299]
*Boesenbergia* *pandurate* extract	*Boesenbergia* *pandurate*	Enhancement of skin moisture, shine, overall elasticity, and reduction in wrinkles.	[300]
Honeybush isolation	*Cyclopia intermedia*	Reduced skin, wrinkle roughness, improved skin hydration levels, and flexibility.	[301]
ɑ-mangostin	*Garcinia mangostana*	Decrease in UV-triggered MMP-1/MMP-9 and wrinkles	[302]
Ferulic acid	*Litchi chinensis*; *Nephelium lappaceum* L.; *Tamarindus indica*	Improving skin elasticity through activities such as bleaching, anti-redness, smoothing, and moisturizing.	[303,304]
Passion fruit seed extract	*Passiflora edulis*	Boosting moisture for dry skin and relieving fatigue.	[298]
Lycopene	*Solanum lycopersicum*	Protects against “erythema” caused by UVB rays and reduces pro-inflammatory cytokines such as “TNF-a” and “IL-6”.	[305]

## Data Availability

All data related to this manuscript are available in the manuscript in the form of tables and figures.
